# Cognitive behavioural therapy for eating disorders: how do clinician characteristics impact on treatment fidelity?

**DOI:** 10.1186/s40337-018-0208-0

**Published:** 2018-09-01

**Authors:** C. E. Brown, K. Nicholson Perry

**Affiliations:** Australian College of Applied Psychology, Discipline of Psychological Science, 255 Elizabeth St, Sydney, NSW 2000 Australia

**Keywords:** Cognitive behavioral therapy, Eating disorders, Knowledge translation strategies, Self-efficacy, Therapist drift, Treatment fidelity

## Abstract

**Background:**

Clinicians routinely report not practising evidence-based treatments with eating disorders. There has been limited research investigating the impact of adaptable clinician characteristics such as self-efficacy and therapeutic optimism in this area. This study evaluated if there is a relationship between clinician therapeutic optimism, self-efficacy and the provision of evidence-based practice in the treatment of bulimia nervosa and binge eating disorder.

**Method:**

A survey developed for this study was administered to 100 psychologists who were recruited online via a range of organisations affiliated with psychology and/or eating disorders. The survey measured demographic factors, eating disorder treatment knowledge, treatment fidelity, the use of individual treatment components and a range of clinician characteristics including self-efficacy and therapeutic optimism.

**Results:**

Results demonstrated that clinician self-efficacy was positively associated with and predicted treatment fidelity. Therapeutic optimism had significant low correlations with treatment fidelity but did not predict treatment fidelity.

**Conclusion:**

These findings would suggest that strengthening clinician self-efficacy is useful in improving evidence-based practice in the treatment of binge eating disorder and bulimia nervosa and may also have implications in the training of clinicians. The study also demonstrated that the use of a range of knowledge translation strategies are valuable in enhancing clinician adherence to evidence-based practice. Further research with direct measures of treatment fidelity is needed to clarify these findings.

**Electronic supplementary material:**

The online version of this article (10.1186/s40337-018-0208-0) contains supplementary material, which is available to authorized users.

## Plain english summary

The best current treatment for bulimia nervosa and binge eating disorder in adults is cognitive behavioural therapy. Despite this, research has shown that many clinicians do not deliver this treatment as intended. There are a range of factors that may impact on why this occurs. In this study, we looked at various clinician characteristics that may impact on the quality of treatment being provided to individuals. It was shown that a clinician’s level of self-efficacy in delivering cognitive behavioural therapy impacted on their ability to implement treatment as planned. This has significant implications in the training of clinicians delivering treatment for adults with eating disorders.

## Background

Bulimia Nervosa (BN) and Binge Eating Disorder (BED) are the two major eating disorders found in those that are not underweight, and defined in the fifth edition of the *Diagnostic and Statistical Manual of Mental Disorders* (DSM-5) [1] by frequent and persistent binge eating episodes. A growing body of research has provided strong empirical support for cognitive behavioural therapy (CBT) based interventions for BN and BED in comparison with psychopharmacology and other types of psychological interventions [2, 3]. A range of psychological therapies have been found to be effective, but findings to date have shown that the most robust and established approach is the use of CBT [2–6]. This is reflected in the recommendation of CBT for the treatment of BN and BED in National Institute for Care and Excellence (NICE) [7] guidelines and most recent version of clinical practice guidelines developed by a multi-disciplinary team with the Royal Australian and New Zealand College of Psychiatrists [8]. Effectiveness studies have also shown that these treatments translate well into *real world* settings for a variety of eating disorders, including BED and BN [9–11]. There is consensus that CBT is an effective evidence-based treatment (EBT) for BED and BN and we need to ensure that the treatment is implemented as intended to ensure the full benefits of treatment are received.

Despite this well-established evidence-base for the efficacy of CBT for BN and BED, a wealth of literature suggests that many psychologists are not delivering treatments as intended (treatment fidelity) and deviating from the protocol over time (therapist drift) [12–15]. Clinicians use an eclectic mix of treatment approaches for these disorders, including unsupported methods [16, 17]. Most individuals reported using multiple theoretical approaches, with only 13% of participants endorsing the use of a single approach [17].

Waller, Stringer and Meyer [12] identified CBT techniques that were essential parts of the protocol for treating eating disorders. In a survey of 80 clinicians, they found that none of these techniques were routinely used [12]. Specifically, clinicians who had high levels of anxiety were more likely to omit exposure-based techniques, such as behavioural experiments, structured eating and completion of food diaries from treatment. Cowdrey and Waller [18] looked at the client perspective and reported their clinicians’ use of eating disorder specific CBT techniques. These included weekly weighing, psychoeducation about eating disorders and how they develop, introducing regular eating, food monitoring records, exposure to feared situations/foods, and concentrating on beliefs about eating/shape/weight in most sessions. These items differentiate treatment from a generic CBT approach and were derived from two evidence-based CBT manuals for the treatment of eating disorders [19, 20]. They found an average of 60.8% reported the use of the eating disorder-specific techniques, with weekly weighing (39.9%) and food monitoring records (53.2%) being used most infrequently. Generic CBT techniques, including agenda setting (29.0%) and surveys/questionnaires (28%) were also used infrequently.

This has also been supported by other studies which collected data directly from clients recalling their experiences of treatment for an eating disorder [18, 21, 22]. One reason for this may be that clinicians do not have the knowledge to implement evidence-based interventions and need to increase their competency in delivering eating disorder treatment to ensure treatment fidelity. Knowledge translation strategies can be described as strategies that augment the translation of research into practice [23]. Knowledge translation strategies that can assist in the delivery of evidence-based practice (EBP) include formal training of clinicians in the treatment protocol, clinical supervision and the use of treatment manuals [24]. These strategies may also, in addition to impacting on clinician behaviour, influence clinician’s attitudes towards delivering the treatment. For example, close supervision may ensure adherence to treatment protocols and quality training may improve their competence in delivering treatment. These strategies simultaneously exposing the clinician to superior client outcomes and influencing their attitude towards the treatment and its potency. There is limited evidence to support this association to date, but this interaction warrants consideration when utilising these strategies.

An alternate explanation could be that clinicians do have the knowledge, but clinician characteristics impact on their delivery of EBP and cause therapist drift [25]. Some clinician characteristics, such as experience, may be anticipated to increase the delivery of EBP. Surprisingly, studies [26, 27] have found that clinician experience does not impact on the effectiveness of EBP. A range of more stable clinician characteristics, such as personality factors, have been found to have a positive impact on treatment fidelity. Both dimensions of openness to experience [28] and openness to feedback [29] have a positive relationship with treatment fidelity.

However they do not represent good targets for intervention due to their stability and there are other, potentially more modifiable characteristics such as clinician anxiety levels, which also seem to impact on a clinician’s ability to deliver evidence-based exposure-based techniques in the treatment of a range of anxiety and eating disorders [12, 30]. Deacon and Farrell [30] believe this to be due to negative clinician beliefs about exposure therapy causing client distress, leading to them to dilute the dose of the treatment protocol. This is despite evidence that suggests exposure-based treatment protocols are well tolerated and acceptable to clients [31]. Furthermore, clinicians with higher levels of depression have been shown to hold more negative attitudes to manualised approaches to treatment and their outcomes [32], believing that a manualised approach negatively impacts the therapeutic alliance.

As the clinician is the primary agent in delivering EBP, exploring specific characteristics that may impact on their ability to change and adapt is useful. The consideration and integration of clinical research, the application of knowledge, and the delivery of EBP may be considered forms of clinician behaviour. If these clinician behaviours are open to change, we can utilise a framework to support clinicians towards behaviours that will improve client outcomes. One of many frameworks within behaviour change theory, such as social cognitive theory (SCT) [33] may inform our understanding of adherence to treatment fidelity through constructs such as self-efficacy and outcome expectancy (optimism). Self-efficacy is central to Bandura’s theory and is the extent to which an individual perceives themselves as capable of completing actions that are required to perform a role effectively [33]. There have been studies in other areas of healthcare that have looked at the positive impact of self-efficacy on delivery of EBP, including dentistry [34] physical therapy [35], medicine [36] and nursing [37]. Furthermore, research in nursing has identified relationships between competence and self-efficacy including its positive influence on a range of factors including career progression, academic motivation, development of skills and learning [38]. Clinicians with greater levels of self-efficacy were discovered to exhibit increased professional behaviour [39].

Therapeutic optimism can be described as a characteristic of the clinician that focuses on outcomes of others in our care [40], and research has explored the positive relationship between optimism and EBP in medicine [36], dentistry [34], mental health workers [41] and nurses [42]. NICE [43] and the Royal Australian and New Zealand College of Psychiatrists [44] guidelines for schizophrenia and other associated disorders suggests that clinicians’ optimistic attitude towards treatment is imperative as it directly impacts patients, carers, and other clinicians. Research has shown that therapeutic optimism is a fluid construct that can be enhanced with education and training [42]. This would support the opportunity for strategies to increase levels of optimism in the delivery of EBT in the field of psychology, including specialist areas such as eating disorders.

These findings warrant further investigation into the clinician characteristics of self-efficacy and therapeutic optimism. Results could be used to guide education, training and support needs for psychologists, specifically to enhance their adherence to EBP and further improve outcomes for clients and reduce the burden on the healthcare system.

The aim of the current study was to examine the relationship between treatment fidelity in CBT for eating disorders and the clinician characteristics of self-efficacy and optimism in a sample of psychologists. Specifically, it was hypothesised that both clinician self-efficacy and therapeutic optimism would be positively associated with treatment fidelity.

## Method

### Participants

The participants were 100 psychologists who all identified as either a provisional (intern), registered or clinical psychologists. For participants to be included in the study, they had to be a provisional, general or clinical psychologist identifying as practicing CBT and to have had a case load that comprises of a minimum of one eating disorder client over the past 12 months. Three psychologists who completed the survey did not meet these criteria and were excluded. The survey was activated by 160 participants of whom: two had not seen an eating disordered client in the past 12 months; one did not utilise CBT; one was a psychiatrist; 17 did not respond to any of the questions; and 39 did not complete the full survey. This resulted in 100 individuals who completed the full survey.

Green [45] provides a thorough overview of methods used to determine sample sizes in regression to ensure adequate power. With the proposed five independent variables, this would give us a sample of size of 90.

### Procedure

An online survey was designed to retrospectively assess clinicians’ self-reported adherence to EBP CBT in treating individuals with eating disorders and obtain additional information regarding a range of clinician characteristics. Participants were psychologists sourced through advertisements, via email, social media and web pages, with a range of organisations including the Butterfly Foundation, Australian Association of Cognitive Behavioural Therapy, Australian and New Zealand Association of Eating Disorders, National Eating Disorder Collaboration, Centre for Eating and Dieting Disorder and eating disorder interest groups. The survey was available for completion from February to July 2017.

Data were gathered via an online survey using Qualtrics. The survey was structured to avoid priming participants, including randomised presentation of questions and they were unable to return to questions to amend answers. The survey consisted of five parts which included demographic and professional information; questions on knowledge translations strategies; self-reported knowledge of CBT for eating disorders; self-reported treatment fidelity for CBT for eating disorders; and clinician self-reported self-efficacy and optimism regarding the delivery of CBT for eating disorders.

Participant demographic variables were collected including age, gender, ethnic background and location. Information related to their professional status was obtained including professional title, options included provisional (intern or higher degree student), registered or clinical psychologist. Other data included provisional pathway to registration, clinical endorsement and location of professional training. Further information was gathered related to their practice in the eating disorder arena such as percentage of current case load of eating disorder clients. Information regarding the clinician’s use of knowledge translation strategies was collected, including attendance at eating disorder-specific training, utilisation of eating disorder-specific supervision and use of a training manual.

### Measures

*Clinician Knowledge* of CBT-based treatment for eating disorders was measured by requesting participants to review one of two modified vignettes that had been used in earlier research [46, 47]. The vignettes gave sufficient information to determine a DSM-5 [1] diagnosis for one of the two eating disorders BN or BED. They were asked what specific CBT-based techniques they would use or not use with this client. Twenty techniques were presented and ranged from eating disorder-specific, generic CBT, or non-endorsed techniques, based on techniques from a study by Cowdrey and Waller [18]. The clinician knowledge score was calculated as an average percentage from self-report of use of the six eating disorder techniques. The overall score ranges between 0 and 100 with high scores reflecting high levels of knowledge and low scores indicating low levels of knowledge regarding CBT treatment for those with BED and BN.

*Treatment fidelity* was the dependent variable and measured by clinican self-rated use of CBT in a clinical setting for BED and BN. The participant was requested to state with what percentage of their clients would they use a range of techniques. The clients were to have presented in the last 12 months and have similar presentation to the one viewed in the vignette (BN or BED). The techniques were as per those utilised for the measure of clinician knowledge, which included eating disorder-specific, generic CBT, or non-endorsed techniques. The treatment fidelity score was derived by taking a mean of the percentage clinicians use the six eating disorder techniques. The overall score ranges between 0 and 100 with high scores reflecting high levels of treatment fidelity and low scores indicating low levels of treatment fidelity.

*Clinician Self-Efficacy* in treating eating disorders was assessed using a modified version of the 10-item Personal Efficacy Beliefs Scale, which was first validated for job-related applications in a work setting [48]. The modifications change the questionnaire to be specific to clinician’s self-efficacy in treating clients with an eating disorder. The Personal Efficacy Beliefs Eating Disorder Scale (PEBS-ED) is self-administered and each participant indicates to what degree they agree or disagree with each item on a 7-point scale from strongly agree (7) to strongly disagree (1). The overall score ranges between 1 and 70 with high scores reflecting high self-efficacy and low scores indicating low self-efficacy. The Cronbach’s α for the PEBS-ED in the present study was 0.88.

*Clinician Therapeutic Optimism* in treating eating disorders was assessed using a modified version of the 10-items Therapeutic Optimism Scale [49]. The modified Therapeutic Optimism Eating Disorder Scale (TOS-ED) was self-administered and based on a 5-point Likert scale ranging from strongly disagree (1) to strongly agree (5). The overall score ranges between 10 and 50 with high scores reflecting high optimism and low scores indicating low optimism. Cronbach’s α for the TOS-ED in the current study was 0.72. See Additional file 1 for copies of the PEBS-ED and TOS-ED.

### Statistical analysis

Bivariate relationships between variables were explored using Pearson and point-biserial correlations. To test the hypothesis that self-efficacy and therapeutic optimism may account for a significant proportion of the variance in clinician treatment fidelity beyond that already accounted for by clinician eating disorder knowledge, a hierarchical multiple regression analysis (MRA) was employed. Treatment fidelity was entered as the dependent variable. To control for the level of knowledge this variable was entered at step 1, followed by the individual predictor variables of self-efficacy and therapeutic optimism at step 2. Only variables that significantly correlated with treatment fidelity were included as predictors in the model. The data were screened for normality and checked for assumption violation. Despite knowledge and treatment fidelity scores being slightly positively skewed when entered into the regression, the residuals were normally distributed and there was no other evidence of multivariate non-normality, non-linearity or heteroscedasticity.

## Results

### Sample demographic variables

The demographic factors of the final sample are shown in Table 1. In total, 100 psychologists met the inclusion criteria and completed the full survey.

**Table 1:** Demographic variables of final sample (*n* = 100) and treatment fidelity correlation

Variable	Final sample	Treatment fidelity correlations^b^
Mean age years (SD)	36.29 (9.04)	−.048
Gender, *n* (%)		–
Female	95 (95)	
Male	5 (5)	
Location, *n* (%)		–
Australia	99 (99)	
New Zealand	1 (1)	
Ethnic background, *n* (%)		–
White/Caucasian	90 (90)	
Other	10 (10)	
Country completed training, *n* (%)		–
Australia & New Zealand	97 (97)	
Other	3 (3)	
Professional Title, *n* (%)^a^		−.227*
Registered Psychologist	39 (39)	
Clinical Psychologist	61 (61)	
Employment status, *n* (%)^a^		.036
Full-time	56 (56)	
Part-time	44 (44)	
Mean Training years (SD)	7.43 (9.49)	.063
Mean eating disordered clients % (SD)	33.11 (33.08)	.435**

**Correlation is significant at the .01 level (two-tailed); *Correlation is significant at the .05 level (two-tailed)

^a^Point-biserial correlation between dichotomous and interval scales. Dichotomous variable coding:

Professional title (Clinical Psychologist = 0, General Psychologist = 1); Employment status (Full-time = 0, Part-time = 1)

^b^Some sample sizes were unequal and therefore unable to permit meaningful statistical comparisons

The sample consisted largely of females in their mid-30s and included an equal proportion of non-higher degree pathway clinicians (general psychologists) and masters/doctorate degree pathway clinicians (clinical psychologists), with fewer trainee psychologists. There were eight trainee psychologists, seven completing the higher degree clinical masters and one the non-higher degree pathway. As no relationship was found between years of training and treatment fidelity, these eight psychologists were assigned to either clinical or general psychologist groups accordingly. There was a range of experience from those who worked solely with eating disorder clients to those that had minimal exposure to this group. Most psychologists had trained and resided in Australia.

### Clinician use of individual techniques in CBT for eating disorders

Table 2 outlines techniques clinicians reported using with clients delivering CBT over the past 12 months with individuals with BN or BED.

**Table 2:** Percentage of clients the clinician reports using the therapeutic techniques as part of their CBT treatment

Technique	Technique used %
Generic CBT techniques	
Demographic variables of final CBT formulation diagram	78.81
Coping in present and future	71.10
Homework tasks	88.24
Looking at links between thoughts, feelings and behaviors	89.37
Thought records	67.86
Thought challenging	80.06
Surveys/Questionnaires	64.57
Behavioral experiments	70.80
Agenda setting	71.21
Mean	75.78
Eating disorder specific CBT techniques	
Concentrated on beliefs about eating/shape/weight in most sessions	63.06
Exposure to feared situations/foods	57.99
Food monitoring records	72.48
Introduce regular eating	76.76
Psychoeducation about eating disorders and how they develop	88.50
Weekly weighing	39.91
Mean	66.40
Unsupported techniques	
Mindfulness	62.08
Schema therapy	21.46
Exploring past/childhood for majority of sessions	18.40
Relaxation techniques	50.49
Motivational interviewing	66.14
Mean	43.71

*n* = 100 for all techniques

There were a range of generic CBT techniques that were used the majority of the time such as looking at links between thoughts, feelings and behaviors, homework tasks, CBT formulation diagrams and thought challenging. Likewise, core components of eating disorder-specific techniques were stated to be used with a large proportion of individuals such as psychoeducation regarding eating disorders and how they develop and the introduction of regular eating. Nevertheless, some evidence-based techniques were less regularly utilised than would be expected, such as exposure to feared situations/foods and weekly weighing. There were also a number of unsupported CBT techniques that were employed at least half of the time, which included relaxation techniques, motivational interviewing and mindfulness.

### Association of clinician characteristics with treatment fidelity

Analysis examining the relationship between professional variables and treatment fidelity are reported in Table 1. There was no relationship found between treatment fidelity and years of training, employment status, or age. As anticipated there was a significant moderate positive correlation between treatment fidelity and percentage of current eating disorder client case load. In addition, there was a small but significant correlation indicating clinical psychologists had higher treatment fidelity scores than general psychologists.

### Relationship between self-efficacy, therapeutic optimism and treatment fidelity

The correlations between clinician treatment fidelity, clinician knowledge, self-efficacy and optimism are presented in Table 3.

**Table 3:** Correlations for treatment fidelity, knowledge, self-efficacy and therapeutic optimism

Variable	Mean (SD)	1	2	3
1. Treatment Fidelity	66.03 (26.62)	–		
2. Knowledge	77.50 (24.20)	.810**	–	
3. Self-Efficacy	46.95 (10.07)	.497**	.357**	–
4. Therapeutic Optimism	42.77 (4.26)	.292**	.210**	.541**

**Correlation is significant at the .01 level (two-tailed)

As predicted, treatment fidelity scores were positively correlated with clinician knowledge (high), self-efficacy (moderate) and therapeutic optimism (low). Results of the MRA are shown in Table 4.

**Table 4:** Hierarchical multiple regression model for variables predicting treatment fidelity

	R^2^	R^2^_change_	Unique variance (%)	B	Se_rb_	*β*	*p*
Step 1^a^	.656	.656					.572
Knowledge				.891	.065	.810	<.001
Step 2^a^	.706	.050					.064
Knowledge			45.7	.797	.065	.724	<.001
Self-Efficacy			3.5	.610	.182	.231	.001
Therapeutic Optimism			0.00	.092	.411	.015	.824

^a^Information regarding the constant was not relevant to the hypothesis therefore omitted

Self-efficacy was positively associated with treatment fidelity. Contrary to expectations, therapeutic optimism had significant low correlations with treatment fidelity but in the MRA model with knowledge and self-efficacy, it did not predict treatment fidelity.

### Relationship between knowledge translation strategies and treatment fidelity

Eighty-seven respondents indicated using one of the three knowledge translation strategies listed, and all three were significantly associated with treatment fidelity (Table 5). Furthermore, the number of these strategies utilised were significantly correlated with both knowledge and treatment fidelity.

**Table 5:** Correlations for sources of knowledge translation strategies (*n* = 100)

	Final sample *n* (%)	Mean knowledge score	Knowledge correlation	Mean treatment fidelity score	Treatment fidelity correlation
Use of training manual^a^			.303^**^		.385^**^
Yes	51 (51)	84.64		76.04	
No	49 (49)	70.07		55.62	
Eating disorder training^a^			. 395^**^		.436^**^
Yes	64 (64)	84.63		74.69	
No	36 (36)	64.81		50.64	
Eating disorder supervision^a^			.419^**^		.396^**^
Yes	57 (57)	86.26		75.15	
No	43 (43)	65.89		53.95	
Number of strategies used			.548^**^		.597^**^
0	13 (13)			37.26	
1	29 (29)			55.87	
2	31 (31)			70.14	
3	27 (27)			86.08	

**Correlation is significant at the .01 level (two-tailed)

^a^Point-biserial correlation between dichotomous and interval scales. Dichotomous variable coding: Use of training manual (No = 0, Yes = 1); attended eating disorder training (No = 0, Yes = 1); attended eating disorder supervision (No = 0, Yes = 1)

Clinician report of how they accessed eating disorder training highlights a large proportion of them accessing training online (30%) or with facilitators from overseas (45%). 37% of clinicians participated in training delivered by Australian facilitators and only 5% had been provided with training in eating disorders as part of their postgraduate university training. Note that several clinicians utilised more than one training method.

## Discussion

### Clinician characteristics of self-efficacy and therapeutic optimism

This study explored the relationship between clinician characteristics of self-efficacy and therapeutic optimism and how they impact on the delivery of EBP in eating disorders. As anticipated our results suggest that increased clinician self-efficacy is associated with higher levels of adherence to EBP. Furthermore, after controlling for clinician knowledge, self-efficacy remained a significant predictor of treatment fidelity.

The hypotheses that therapeutic optimism would be associated with treatment fidelity was not confirmed. One possible explanation for this is that a moderate correlation was identified between the two variables self-efficacy and therapeutic optimism. When entered into a MRA, there was a significant amount of shared, rather than unique, variance and self-efficacy had a greater exclusive influence on treatment fidelity than therapeutic optimism. It may be that therapeutic optimism alone will not produce high levels of treatment fidelity if the core competencies (knowledge) and/or self-efficacy are lacking. Thus, the measure of therapeutic optimism is primarily related to the clinician’s hopes for positive treatment outcomes rather than with their own sense of proficiency of the treatment protocol. It is possible that once a clinician reaches a level of self-efficacy, their degree of therapeutic optimism moderates the relationship between self-efficacy and treatment fidelity. The current findings are partially consistent with the view of Bandura [33], in that self-efficacy is a predictor of behaviour change (clinician adherence to treatment fidelity), however the contribution of outcome expectancy (therapeutic optimism) was not supported.

One important finding relates to the training of clinicians in the area of eating disorders. Strengthening clinician self-efficacy appears to contribute to improving evidence-based practise in the treatment of BED and BN. By identifying a clinician’s level of self-efficacy via the use of a specific tools, such as the PEBS-ED utilised in this study, supervisors could tailor their support to develop a personalised training plan promoting the effective delivery of EBTs by their supervisees. For example, the support provided to those with high levels of self-efficacy may be limited to guidance around managing challenging clients or atypical presentations. Conversely, those with low self-efficacy may require considerable support, intensive training, access to a formal protocol, as well as regular supervision which may involve observation, role playing and co-therapy, in order to increase their sense of self-efficacy especially in delivering specific strategies they may question or feel anxious administering.

### Clinician use of individual techniques in CBT for eating disorders

Consistent with previous findings [12, 13] core elements of eating disorder treatment were used much less regularly than would be expected. Of 100 responders, they reported using eating disorder-specific techniques with an average of 66.4% of their clients during treatment. Those that were exposure-based were utilised less frequently, with only 42% of clinicians exposing clients to feared situations/foods. 64.8% of these reported this technique was not appropriate due to dual diagnosis, complex presentation, they considered the technique ineffective, or they did not believe it was an essential part of treatment. Similarly, only 39.91% of their clients were engaging in weekly weighing as part of their protocol, 44.2% of clinicians stating similar reasons for not using this technique. Clinician’s attitudes towards these techniques may impact on the dose of treatment being administered and therefore treatment outcomes for clients. Furthermore, unsupported techniques such as mindfulness, motivational interviewing and relaxation strategies were used over 50% of the time.

The research differs considerably to these clinician beliefs, suggesting that clinicians can effectively deliver a highly manualised treatment protocol to those with severe mental illness and comorbidity, without compromise to the structured sessions or impact on the therapeutic relationship [50, 51]. The negative attitudes of clinicians in the use of EBT manuals can predict poorer outcomes for clients [52]. A recent study has shown that even brief interventions (90 min) can have a significant impact, improving clinician attitudes towards exposure therapy [53]. Additionally, [54] research has demonstrated that training focussing specifically on addressing clinician concerns via a variety of methods during training not only reduced their concerns regarding delivery of exposure based techniques, but also increased self-reported use of the procedure. This would support the notion that clinician characteristics can have a significant impact on not only the delivery of treatment protocols but also, and more importantly, on the treatment outcome for the client. This also highlights the role that knowledge translations strategies could have on challenging these clinician beliefs.

### Knowledge translation strategies and treatment fidelity

Strategies for improving clinician delivery of EBP include formal training of clinicians in treatment protocols, clinical supervision and the use of treatment manuals [24]. Manuals and formal training can provide the foundation of learning in psychological interventions. Consistent with previous research [12, 17, 32] treatment manuals were only utilised by 51% of clinicians in this study.

Eating disorder-specific training for BN and BED was utilised by 64% of clinicians. Interestingly 30% of those had accessed online training; 45% attended training with international professionals; 37% with Australian clinicians, with only 5% reported completing eating disorder training as part of their postgraduate study. An important issue emerging from these findings is the apparent lack of training opportunities in CBT for eating disorders in Australia during postgraduate study, and post registration as a psychologist. This is consistent with prior findings that despite there being a significant demand for training in psychological treatment practices, access to specialist training and supervision can be challenging and expensive [55].

Manuals and short training courses are helpful but fall short of the treatments used in randomised controlled trials, where regular and intensive supervision supplements training. This emphasises the importance of not only didactic training and guidance through a formal protocol and manual, but also regular supervision by a qualified clinician specialising in eating disorders who can assist to translate this knowledge into the delivery of EBP. Surprisingly, data revealed that only 57% of clinicians were accessing supervision to support their work with eating disordered clients. This is concerning especially given that standards for maintaining registration as a psychologist in Australia require a minimum number of supervision hours per year. Outcomes from this study demonstrate that all three of these strategies; clinical supervision, use of a manual and training, are helpful in improving treatment fidelity in CBT for eating disorders. There were no particular combinations or individual strategies that were superior, but results showed that the more strategies utilised, the higher the clinician adherence to treatment fidelity. Supervision may be the one strategy with an individual, interactive framework that can assist in determining clinician self-efficacy, setting suitable personalised goals and identifying strategies to improve clinician deficits in delivering EBP.

It is suggested that the optimal environment to utilise a range of knowledge translation strategies is University training at postgraduate level, where theoretical models and formulation are incorporated into the delivery of treatment within internal or external clinical placements. We know that the scientist practitioner model is utilised in the majority of training institutions for clinical psychology in Australia [56]. It would be interesting to determine how many institutions specifically train students in the delivery of EBP for eating disorders and how this is provided to prepare individuals for engaging with this client group at the onset of their career.

### Limitations and future research

There are some limitations to the current study which should be considered. One main limitation is that the study assessed self-report of treatment delivered retrospectively over the past 12 months, with a varying number of eating disorder clients; an indirect measure of treatment fidelity. It would be important for future studies to focus on obtaining direct measures of treatment fidelity to reduce bias and subjectivity of answers.

Secondly, whilst the scales used to evaluate self-efficacy and therapeutic optimism were based on other scales validated in studies of other healthcare providers, they have not been used in previous research. Further validation of these scales with a psychologist population would be an important focus for future research.

An extension of the literature on self-efficacy may include examining its relationship with treatment fidelity with other therapies in a range of mental health conditions where there is evidence of therapist drift, to determine if the findings of this study apply and extend to other areas of clinical work in psychology. A diverse range of healthcare clinicians deliver CBT for eating disorders including occupational therapists, psychiatrists, mental health nurses and social workers. It would be pertinent to explore outcomes for a range of professions and how they relate to the different professional training frameworks. Future research extending our understanding of other clinician characteristics amenable to change and that impact on delivery of EBTs, would be useful and could be targeted as part of supervision or personal development.

As findings show that clinicians are not utilising exposure-based techniques in CBT-based therapies across a range of disorders, future dismantling studies would be valuable in determining which specific components of CBT for eating disorders are most effective and the essential components of treatment. A recent systematic review and meta-analysis [57] has shown with brief intensive and concentrated CBT treatments for anxiety disorders, that incorporate the fundamental technique of exposure, produce similar treatment outcomes to standard full dose CBT interventions. Due to the shared comorbidity and mechanisms underpinning anxiety and eating disorders, it is anticipated such exposure-based techniques may be imperative in the treatment of eating disorders. There is currently research underway to explore the impact of brief intensive CBT-based interventions in eating disordered outpatients, to explore the core components of the treatment essential for obtaining optimal treatment outcomes [58].

It would be important to survey Australian institutions to determine what elements of eating disorder training, are incorporated into training programs. The postgraduate years would certainly be an optimum time to expose early career clinicians to the area of eating disorders where they have access to a range of knowledge translation strategies as part of a structured evidence-based program, giving rise to a greater number of quality treatment options in the community.

## Conclusion

Improving clinician self-efficacy appears useful in improving evidence-based practice in the treatment of BED and BN. These findings may have implications and be an important consideration in the training of clinicians. Knowledge translation strategies such as supervision, training and the use of manuals are valuable in enhancing clinician adherence to evidence-based practice. Further research with direct measures of treatment fidelity is needed to clarify these findings.

## Additional file

Additional file 1: Questionnaires: PEBS-ED and TOS-ED. (PDF 89 kb)

## Abbreviations

*AN*: Anorexia nervosa
*BED*: Binge eating disorder
*BN*: Bulimia nervosa
*CBT*: Cognitive behavioural therapy
*DSM-5*: Diagnostic and statistical manual of mental disorders, fifth edition
*EBP*: Evidence-based practice
*EBT*: Evidence-based treatment
*MRA*: Multiple regression analysis
*NICE*: National Institute for Healthcare and Excellence
*PEBS-ED*: Personal efficacy beliefs eating disorder scale
*SCT*: Social cognitive theory
*TOS-ED*: Therapeutic optimism eating disorder scale
