# Supplementary material for: Cognitive behavioural therapy for eating disorders: how do clinician characteristics impact on treatment fidelity?
Source: J Eat Disord. 2018 Sep 1;6:19. doi: 10.1186/s40337-018-0208-0 (PMC6119328; doi:10.1186/s40337-018-0208-0)
Supplement: Supplementary file 1 — Questionnaires: PEBS-ED and TOS-ED. (PDF 89 kb) [file 40337_2018_208_MOESM1_ESM.pdf]

Personal Efficacy Beliefs Eating Disorder Scale (PEBS-ED)

Below you will find a series of statements. Please read each statement and indicate to what extent the statement in true of you by endorsing the number that is closest using the scale below. Remember, there are no right or wrong answers.

1<br>strongly disagree	2<br>mostly disagree	3<br>somewhat disagree	4<br>neutral	5<br>somewhat agree	6<br>mostly agree	7<br>strongly agree

1. I have confidence in my ability to treat clients with eating disorders. __________
2. There are some tasks required when treating clients with eating disorders that I cannot do well. * __________
3. When my performance is poor, it is due to my lack of ability. * __________
4. I doubt my ability to treat clients with eating disorders. * __________
5. I have all the skills needed to perform my job very well. __________
6. Most clinicians who treat clients with eating disorders can do this job better than I can. * __________
7. I am an expert in treating eating disorders. __________
8. My future in eating disorders is limited because of my lack of skills. * __________
9. I am very proud of my skills and abilities in treating eating disorders. __________
10. I feel threatened when others watch me work. * __________

Total PEBS-ED score = __________

Scoring

* Items marked with an asterisk were reverse scored.

The Personal Efficacy Beliefs Eating Disorder Scale (PEBS-ED) is a modified version of the 10-item Personal Efficacy Beliefs Scale, which was first validated for job-related applications in a work setting (Riggs, Warka, Babasa, Betancourt, & Hooker, 1994). The PEBS-ED is self-administered, and each participant indicates to what degree they agree or disagree with each item on a 7-point scale from strongly agree (7) to strongly disagree (1). The overall score ranges between 1 and 70 with high scores reflecting high self-efficacy and low scores indicating low self-efficacy.

Reference

Riggs, M. L., Warka, J., Babasa, B., Betancourt, R., & Hooker, S. (1994). Development and validation of self-efficacy and outcome expectancy scales for job-related applications. *Educational and Psychological Measurement, 54*(3), 793. doi:10.1177/0013164494054003026

Therapeutic Optimism Eating Disorder Scale (TOS-ED)

Below you will find a series of statements. Please read each statement and indicate to what extent the statement is true of you by endorsing the number that is closest using the scale below. Remember, there are no right or wrong answers.

1	2	3	4	5
strongly disagree	somewhat disagree	neutral	somewhat agree	strongly agree

1. Mental health clinicians have the capacity to positively influence outcomes for people with eating disorders. __________
2. There is little that can be done to help many people with eating disorders. * __________
3. My contribution to positive outcomes is insignificant in comparison to other treatments, for example, medications. * __________
4. I can make a positive difference to outcomes for most people with eating disorders. __________
5. Positive outcomes are directly related to the quality of eating disorder clinician skills and knowledge. __________
6. There are always new skills and knowledge I can acquire to improve my work. __________
7. The outcome of eating disorder treatment is not significantly affected by clinician interventions. * __________
8. Even the most challenging clients can benefit from my intervention. __________
9. Often there is little I can do to help people with their eating disorders. * __________
10. With my assistance most people with eating disorders will recover. __________

Total TOS-ED score = __________

Scoring

* Items marked with an asterisk were reverse scored.

The Therapeutic Optimism Eating Disorder Scale (TOS-ED) is a modified version of the 10-items Therapeutic Optimism Scale (Byrne, Sullivan, & Elsom, 2006). This scale was first validated with mental health workers to determine how clinician optimism impacted on client outcome. The scale has previously been modified for use in nursing samples (Elsom & McCauley-Elsom, 2008a, 2008b), and more recently for use with drug and alcohol clinicians (Best, Savic, & Daley, 2016). The TOS-ED is self-administered and based on a 5-point Likert scale ranging from strongly disagree (1) to strongly agree (5). The overall score ranges between 10 and 50 with high scores reflecting high optimism and low scores indicating low optimism.

References

Best, D., Savic, M., & Daley, P. (2016). The well-being of alcohol and other drug counsellors in Australia: Strengths, risks, and implications. *Alcoholism Treatment Quarterly, 34*(2), 223-232. doi:10.1080/07347324.2016.1148514

Byrne, M. K., Sullivan, N. L., & Elsom, S. J. (2006). Clinician optimism: development and psychometric analysis of a scale for mental health clinicians. *Australian Journal of Rehabilitation Counselling, The, 12*(1), 11-20. doi:10.1375/jrc.12.1.11

Elsom, S., & McCauley-Elsom, K. (2008a). Mental health nurses' therapeutic optimism: a tale of two conferences. *International Journal of Mental Health Nursing., 17*(1), A7.

Elsom, S., & McCauley-Elsom, K. (2008b). Measuring therapeutic optimism. *Australian and New Zealand Journal of Psychiatry., 42*(3), A51.
